# A Novel Re-Entrant Honeycomb with Tunable Auxetic Response and Enhanced In-Plane Mechanical Performance

**DOI:** 10.3390/ma19163533

**Published:** 2026-08-20

**Authors:** Chenxi Li, Siyan Bai, Jueran Yin, Qianqi Cao, Haoxin Tian, Yifei Dong, Han Wang, Jiurui Liu, Guocheng Que, Yiwen Chen

**Affiliations:** School of Intelligent Manufacturing, Jiangnan University, Wuxi 214122, China

**Keywords:** re-entrant honeycomb, X-shaped ligament, cosine-curved wall, in-plane compression, energy absorption

## Abstract

Re-entrant honeycombs with tunable auxetic response have attracted increasing attention for energy absorption, yet their in-plane stiffness and load-bearing capacity are often limited by bending-dominated deformation and local instability. In this study, a re-entrant honeycomb with cosine-curved walls and X-shaped ligaments, denoted as X-CRS, was proposed to enhance the in-plane mechanical response and energy absorption performance. Three X-CRS configurations with different X-ligament angles were fabricated by fused deposition modeling using PLA and investigated through quasi-static compression tests and finite element simulations. The results show that the X-CRS-30 achieved the highest load-bearing performance, with approximate increases of 148%, 133%, and 102% in elastic modulus, collapse stress, and plateau stress, respectively, and the highest mean specific energy absorption (SEA) of 1.225 ± 0.015 kJ kg^−1^. At a nominal compressive strain *ε*_c_ = 0.20, the numerical effective Poisson’s ratios *ν*_eff_ were −0.676, −0.609, −0.376, and +0.156 for CRS, X-CRS-10, X-CRS-20, and X-CRS-30, respectively. Thus, X-CRS-20 retained auxeticity with more stable progressive collapse, whereas X-CRS-30 prioritized stiffness and energy absorption at the expense of auxetic response.

## 1. Introduction

Auxetic honeycombs have attracted sustained interest in lightweight protective and energy-absorbing structures because they can provide enhanced energy dissipation, crushing resistance, and deformation controllability under compressive loading [1,2,3,4]. Unlike conventional honeycombs, which generally expand laterally under in-plane compression, auxetic honeycombs contract in the transverse direction and therefore exhibit an effective negative Poisson’s ratio. This response is governed primarily by the geometry of the unit cell rather than by the intrinsic properties of the constituent material. Among the various auxetic configurations, the re-entrant honeycomb is one of the most representative designs. Its inwardly inclined cell walls promote ligament bending and joint rotation during compression, enabling the unit cells to contract laterally and providing a simple geometric route for transforming conventional hexagonal honeycombs into auxetic cellular structures [5,6,7].

Despite their geometric simplicity and auxetic capability, conventional re-entrant honeycombs usually suffer from relatively low in-plane stiffness and strength [8,9,10]. Their compressive response is mainly governed by cell-wall bending, joint rotation, and local buckling, which restrict the formation of stable load-transfer paths and limit the attainable energy absorption capacity. To overcome these limitations, extensive architectural modifications have been introduced, including gradient layouts [11,12], hierarchical configurations [13,14], curved ligaments [15], helical-wall architectures [16,17], embedded units [18,19], and hybrid topologies [20]. These strategies can redistribute deformation, delay localized collapse, and improve crushing resistance. However, stiffness enhancement and auxetic deformation are often competitively coupled in re-entrant structures [21]. Reinforcing members may increase the initial resistance to compression and improve load-bearing capacity, but excessive constraint can suppress transverse contraction and weaken the negative Poisson’s-ratio response [22,23,24]. Therefore, an effective design should not only introduce additional load-transfer paths, but also preserve sufficient deformation compatibility among the curved cell walls, reinforcing ligaments, and rotating unit-cell components [25,26].

This trade-off suggests that the mechanical enhancement of re-entrant honeycombs should be achieved through coordinated deformation regulation rather than simple structural reinforcement. Curved cell walls [15,27] can modify the bending path and delay localized instability, while X-shaped ligaments [28] can provide additional load-transfer routes within the unit cell. Their combination therefore offers a potential route to improve in-plane stiffness, crushing resistance, and energy absorption while retaining a tunable auxetic response. Unlike curved-ligament designs that primarily reshape the deformation trajectory, and unlike internally reinforced or hybrid re-entrant topologies that mainly increase structural constraint, X-CRS combines cosine-curved re-entrant walls with an angle-controlled X-shaped load-transfer path within the same unit cell.

Accordingly, this study investigates a curved-wall X-reinforced re-entrant honeycomb (X-CRS) through quasi-static compression experiments and finite element simulations. The stress–strain response, deformation mode, energy absorption, and Poisson’s-ratio evolution are evaluated to determine how the X-ligament angle regulates the competition between re-entrant-wall rotation and ligament-dominated load transfer. This framework clarifies the resulting balance among auxeticity, collapse stability, stiffness, and energy absorption.

## 2. Materials and Methods

### 2.1. Design of Specimens

**Figure 1:**
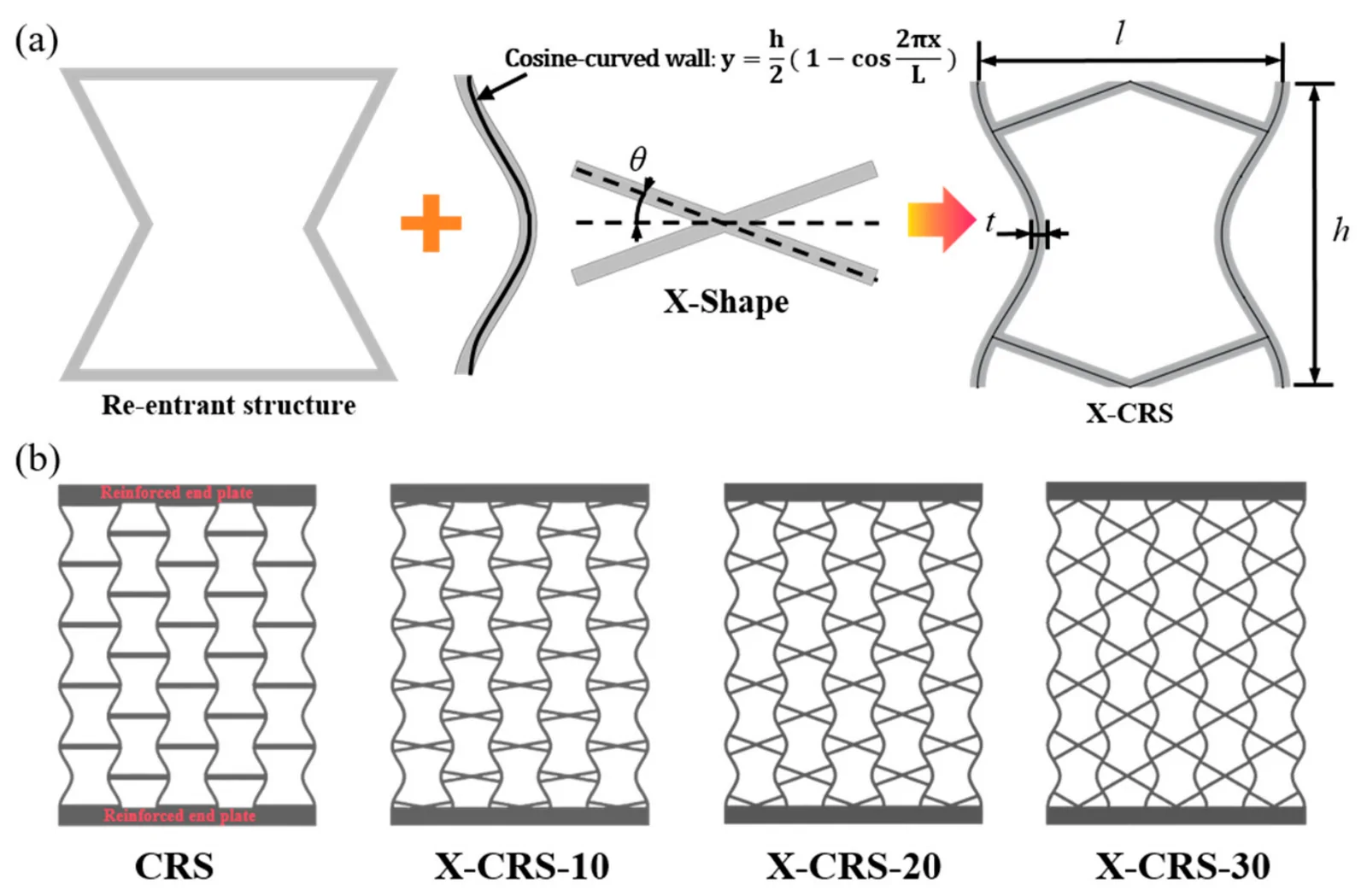
The schematic design process of X-CRS: (**a**) the unit cell design; (**b**) the overall structures of CRS and X-CRS-10/20/30.

As shown in Figure 1a, the proposed honeycomb was derived from a conventional re-entrant configuration by replacing the straight inclined walls with cosine-curved re-entrant walls and reconstructing the horizontal member into an X-shaped ligament connection. The unit-cell geometry is characterized by ligament length (*l*), cell height (*h*), X-ligament angle (*θ*), and wall thickness (*t*). For each X-CRS configuration, the X-ligament centerlines pass through the unit-cell center *O*(0, 0) at ±*θ*, and the attachment points are defined by their intersections with the centerline of the curved wall; the corresponding coordinates and full ligament lengths are listed in Table 1. Three configurations (X-CRS-10, X-CRS-20, and X-CRS-30) were constructed as 5 × 5 cell arrays. Reinforced top and bottom end plates with *T* = 6 mm were used to reduce boundary-induced premature buckling, as shown in Figure 1b.

The X-ligament angle *θ* was the only varied geometric parameter; the curved-wall geometry, wall thickness, specimen dimensions, and cell array were kept unchanged. The selected values of 10°, 20°, and 30° span the low, intermediate, and high portions of the investigated range and were chosen to reveal the angle-dependent trend and possible transition.

### 2.2. Specimen Preparation and Experimental Setup

**Figure 2:**
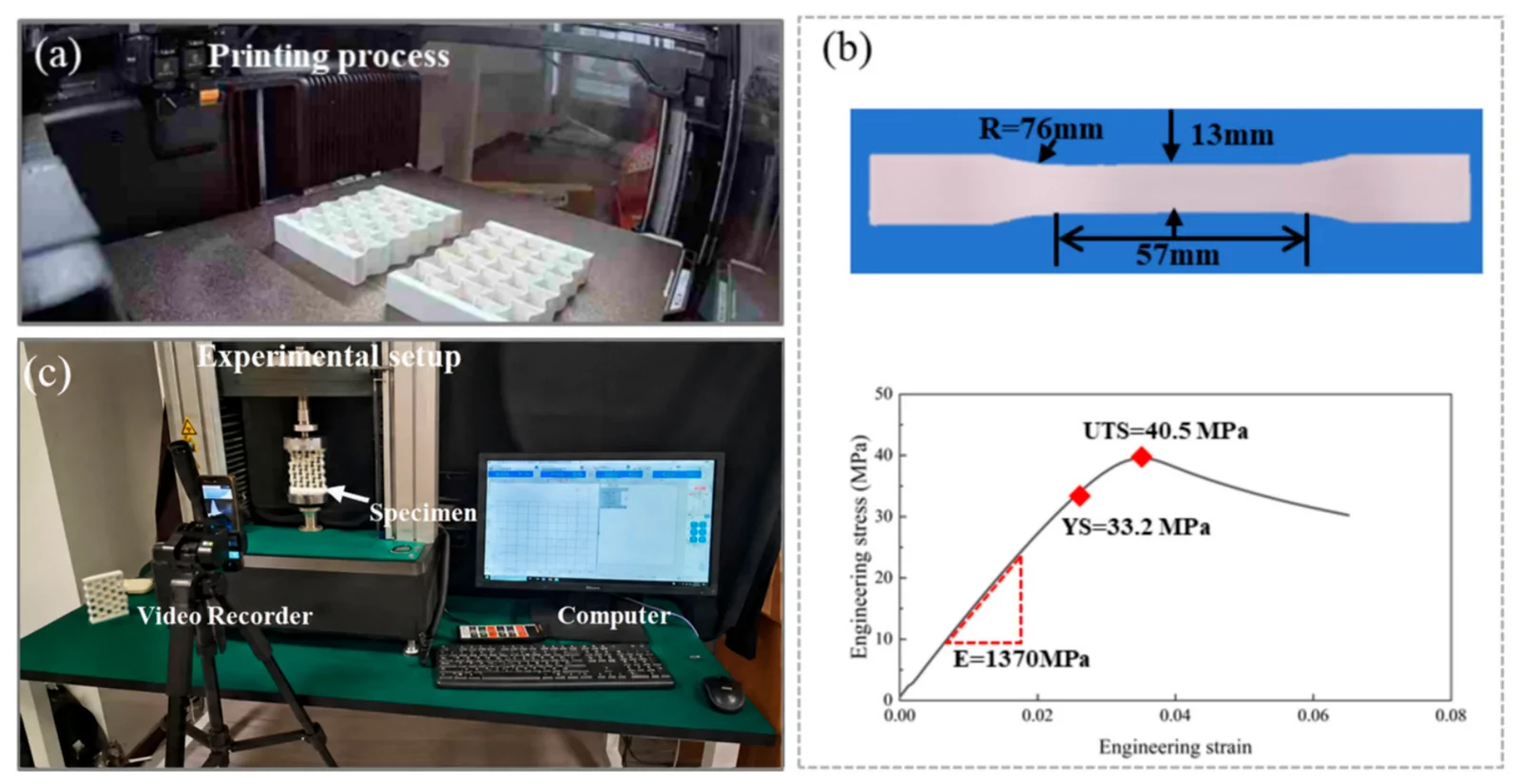
Specimen preparation and experimental setup: (**a**) printing process; (**b**) tensile-coupon geometry and measured PolyMax PLA response; (**c**) in-plane compression setup.

All honeycomb specimens were fabricated by fused deposition modeling (FDM) using commercial PolyMax PLA filament (Polymaker, Changshu, China), as shown in Figure 2a. Tensile coupons were printed using the same processing parameters; the coupon geometry and measured response are shown in Figure 2b, and the measured elastic modulus, yield strength, and ultimate tensile strength were 1370, 33.2, and 40.5 MPa, respectively. In-plane compression tests used a 10 kN load cell and two parallel steel platens of 118 mm diameter. Before each test, the specimen center was aligned with the platen center. Specimens were compressed at 6 mm min^−1^, corresponding to a nominal strain rate of 0.001 s^−1^. Both the upper and lower faces of each specimen were in unbonded dry frictional contact with the steel platens. Nominal strain and stress were calculated as *ε* = *δ*/*H*_0_ and *σ* = *F*/*A*_0_, respectively, where *H*_0_ = 100 mm and *A*_0_ = 85 mm × 20 mm. The deformation was recorded by video, and three repeated tests were conducted for each configuration.

In the adopted slicing strategy, shell thickness was jointly defined by the line width, number of wall loops, and top/bottom solid-layer settings in Table 2 rather than by one independent parameter. The specimens were printed flat, with the honeycomb plane parallel to the build plate and the layer-stacking direction through the specimen thickness.

### 2.3. Numerical Simulation and Validation

Abaqus/Explicit 2024 (Dassault Systèmes SIMULIA Corp., Providence, RI, USA) was used to simulate in-plane compression with a moving rigid platen, a fixed rigid platen, and a honeycomb specimen discretized using C3D8R elements with reduced integration and hourglass control. General Contact with ALL EXTERIOR included platen–specimen contact and wall–wall self-contact; hard normal contact and Coulomb friction with *μ* = 0.10 were specified. PolyMax PLA was represented by an effective isotropic elastoplastic model with *E* = 1370 MPa, *ν* = 0.30, *ρ* = 1240 kg m^−3^, and coupon-derived plastic hardening. The upper platen was prescribed to *U*_3_ = −100 mm over 0.1 s; no artificial mass scaling was used. Post-test inspection of the highly deformed CRS and X-CRS-30 specimens showed no visible macroscopic cracking or ligament fracture (Supplementary Figure S3a,b). Therefore, damage evolution was not included. For every configuration, *ν*_eff_ was calculated from 16 geometrically corresponding nodes forming eight transverse pairs in the cellular gauge region.

**Figure 3:**
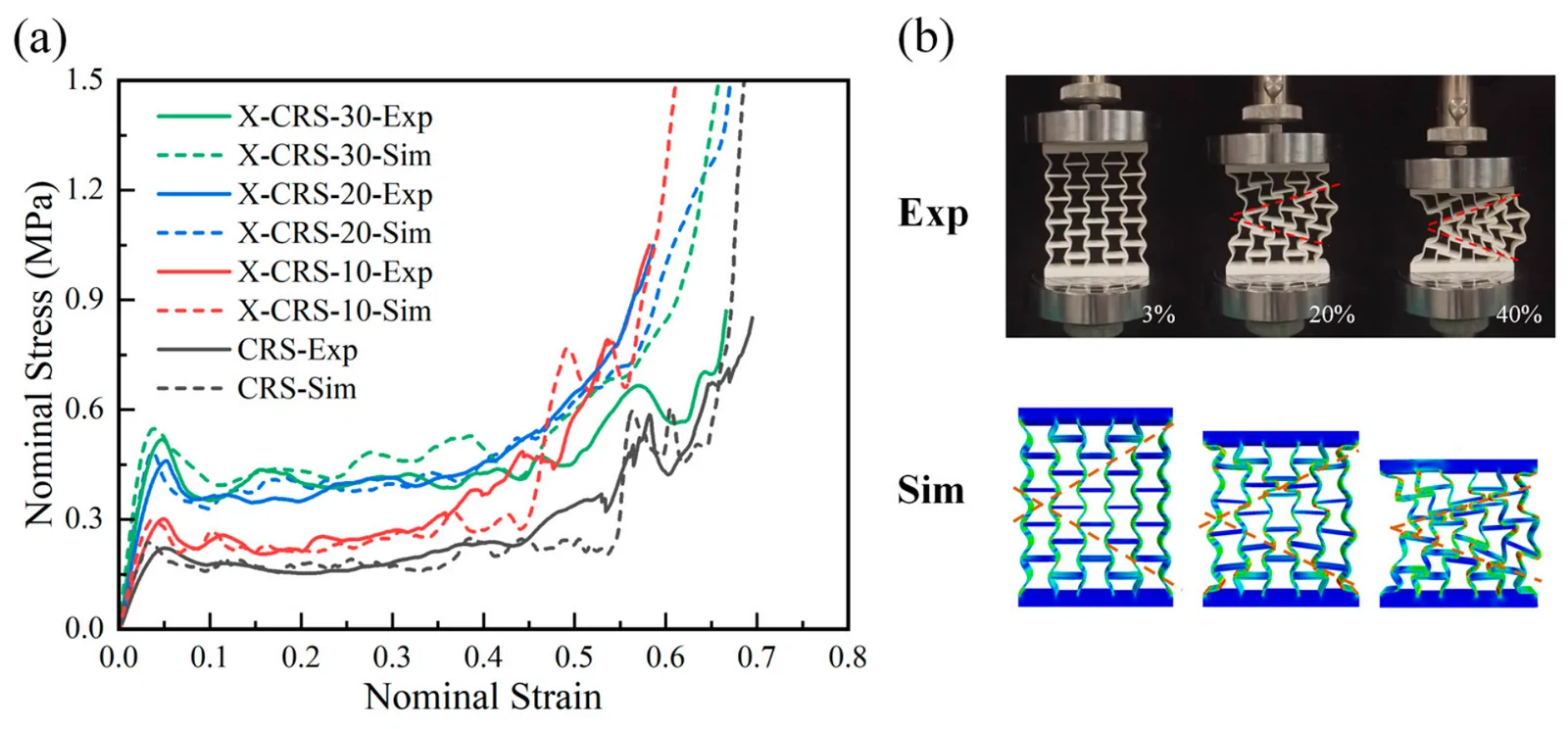
Finite element model validation: (**a**) experimental and simulated nominal stress–strain curves; (**b**) experimental and simulated CRS deformation patterns at representative nominal compressive strains. Nominal strain is dimensionless (mm/mm).

The simulated nominal stress–strain curves were compared with the experiments in Figure 3a, and CRS was used as a representative case for the deformation comparison in Figure 3b. The model reproduces the overall response, principal deformation stages, and configuration-dependent trends, although individual peaks and local fluctuations are not matched point by point. The key finite element settings and verification results are summarized in Table 3.

Mesh sensitivity was assessed using global element sizes of 0.50, 0.33, and 0.25 mm (Supplementary Figure S1). Relative to the 0.25 mm reference, the 0.33 mm production mesh differed by 3.68% in full-curve *L*_2_ norm, 1.80% in collapse stress, 1.54% in plateau stress, 0.81% in SEA, 2.31% in densification strain, and 5.96% in the initial-modulus diagnostic. The X-CRS-20 *ν*_eff_ curve differed by 2.46% between the 0.33 and 0.25 mm meshes; therefore, 0.33 mm was adopted.

The quasi-static character of the Explicit simulations was assessed using ALLKE/ALLIE (Supplementary Figure S2). Over 0.02 ≤ *ε* ≤ 0.60, the maximum ratios were 5.69%, 8.08%, 2.23%, and 2.37% for CRS, X-CRS-10, X-CRS-20, and X-CRS-30, respectively; after *ε* = 0.05, all ratios remained below 1.6%.

### 2.4. Performance Metrics

To quantify mechanical performance and energy absorption, characteristic indicators were extracted from the nominal stress–strain curves. The elastic modulus (*E*) was obtained from the initial linear region, while collapse stress (*σ*_c_) was defined as the first peak preceding progressive collapse. Plateau stress (*σ*_pl_), which represents the average load-bearing capacity during stable crushing, was calculated as follows:

(1) $$\sigma_{pl}=\frac{1}{\varepsilon_{d}-\varepsilon_{cp}}\int_{\varepsilon_{cp}}^{\varepsilon_{d}}\sigma \left(\varepsilon \right)d\varepsilon$$

where *ε*_cp_ is the strain corresponding to the first collapse peak *σ*_c_, and *ε*_d_ is the densification strain.

The densification strain (*ε*_d_) was determined using the energy-absorption-efficiency criterion:

(2) $${\left.\frac{d}{d\varepsilon }\left[\frac{1}{\sigma \left(\varepsilon \right)}\int_{0}^{\varepsilon }\sigma \left(\varepsilon \right)d\varepsilon \right]\right|}_{\varepsilon =\varepsilon_{d}}=0$$

The post-collapse global maximum of the energy-absorption efficiency was identified computationally, and the corresponding strain was taken as *ε*_d_; the same criterion was applied to every experimental curve. Absorbed energy per unit volume (*W*) was then obtained by integrating the stress–strain response up to a given strain:

(3) $$W=\int_{0}^{\varepsilon }\sigma \left(\varepsilon \right)d\varepsilon$$

Specific energy absorption (SEA) was calculated by normalizing absorbed energy by structural density:

(4) $$SEA=\frac{W}{\rho }$$

For statistical comparison, *E*, *σ*_c_, *σ*_pl_, *ε*_d_, and SEA were extracted from the three replicate tests for every configuration and are reported as mean ± sample standard deviation.

## 3. Results and Discussion

### 3.1. In-Plane Mechanical Response and Energy Absorption

**Figure 4:**
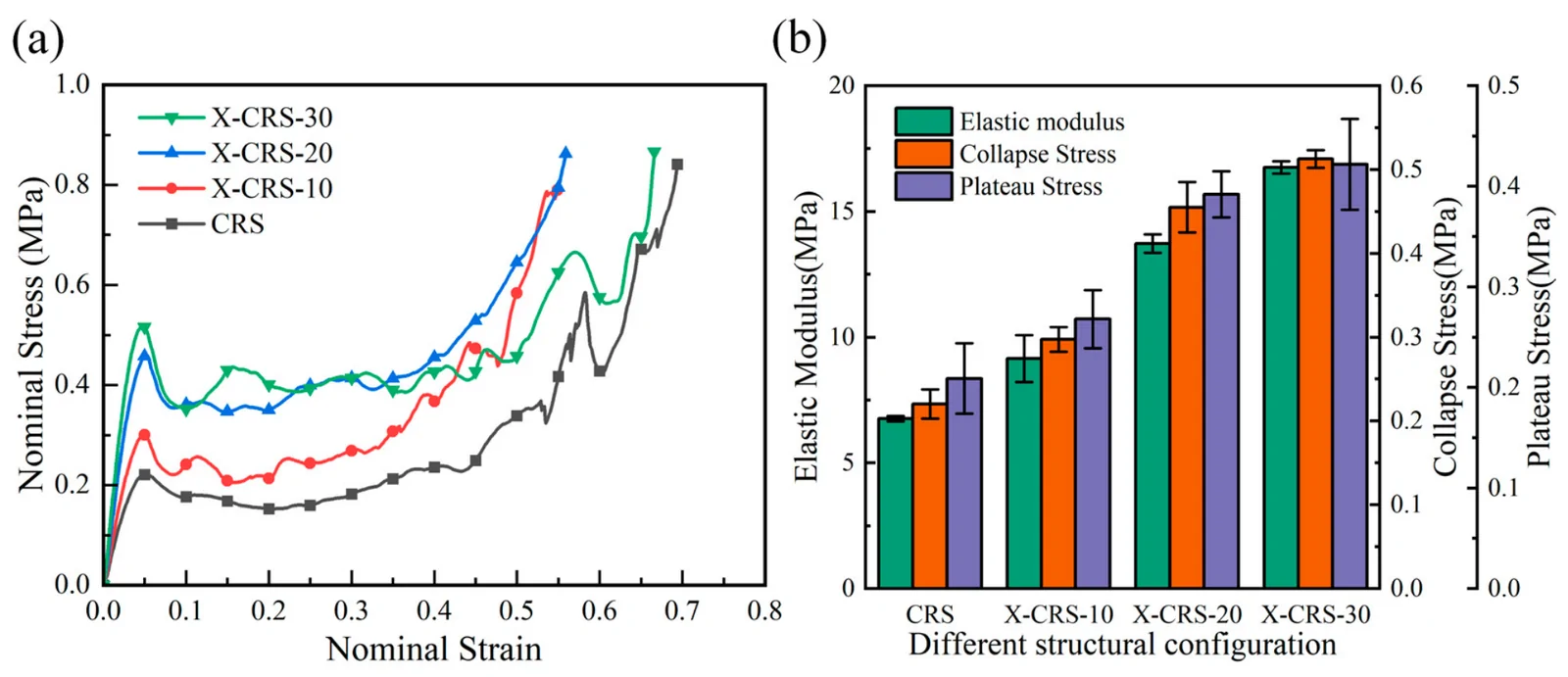
Experimental mechanical response under quasi-static in-plane compression: (**a**) group-mean nominal stress–strain curves (*n* = 3); (**b**) elastic modulus, collapse stress, and plateau stress. Error bars denote sample standard deviations.

Figure 4a presents the nominal stress–strain responses under quasi-static in-plane compression. All configurations show an initial elastic stage, a plateau stage dominated by bending, rotation, and local collapse, and densification caused by extensive wall contact. Figure 4b compares the principal mechanical indicators: X-CRS-30 reached *E* = 16.76 ± 0.243 MPa, *σ*_c_ = 0.512 ± 0.010 MPa, and *σ*_pl_ = 0.422 ± 0.045 MPa, approximately 148%, 133%, and 102% above CRS. Nominal stress *σ* = *F*/*A*_0_ is used as an architecture-level measure; local numerical stresses are interpreted against the measured PolyMax PLA properties. Increasing *θ* therefore strengthens the internal load-transfer path and improves initial stiffness and plateau capacity.

With *n* = 3 per configuration, exploratory group effects were evaluated using exact-permutation Kruskal–Wallis tests. Significant overall group effects were detected for *E*, *σ*_c_, *σ*_pl_, and SEA (*p* ≤ 0.0069), whereas *ε*_d_ did not reach the 0.05 significance level (*p* = 0.0599). Because the Kruskal–Wallis test evaluates overall group effects rather than specific pairwise differences, the percentage changes between individual configurations are reported descriptively.

**Figure 5:**
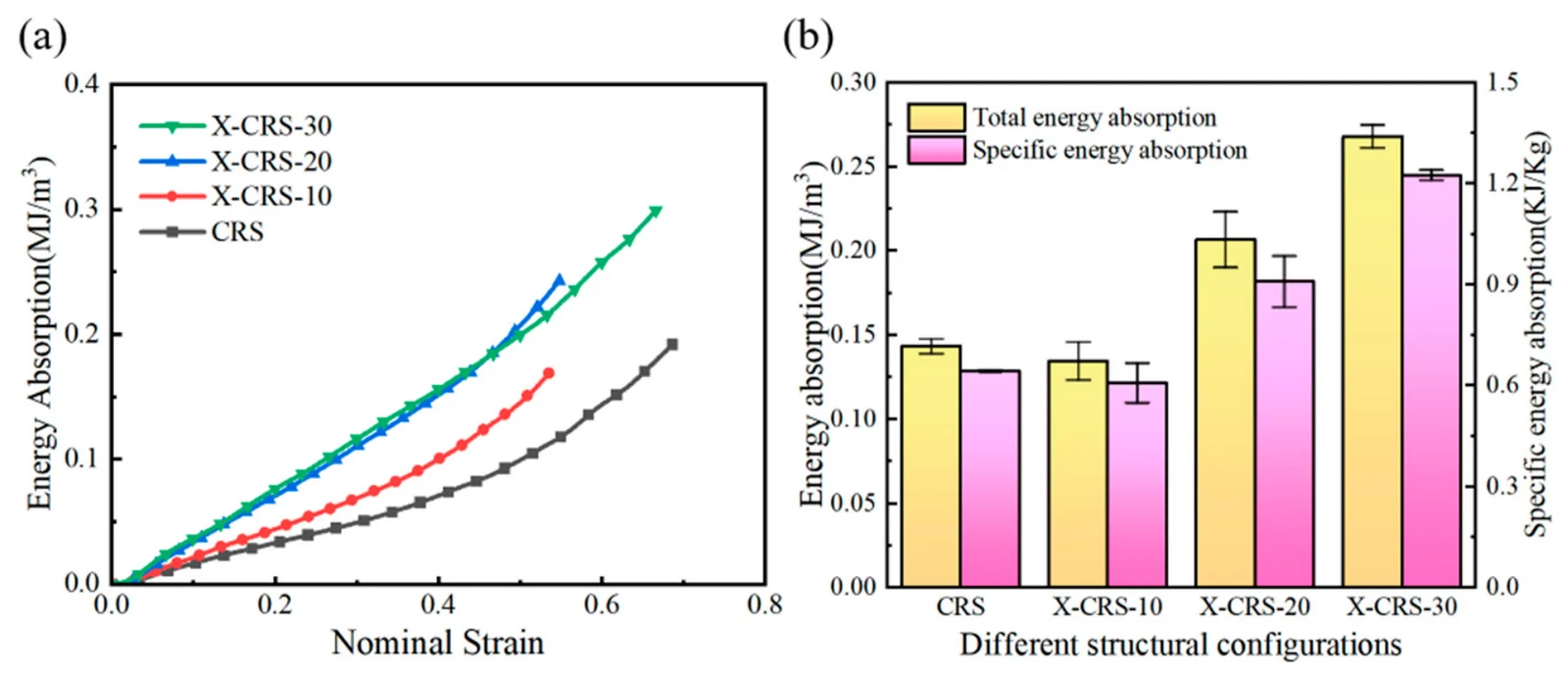
Energy-absorption performance: (**a**) response before densification; (**b**) comparison of absorbed energy and SEA.

Figure 5a shows the energy-absorption response before densification. X-CRS-20 and X-CRS-30 reach higher final absorbed energy than CRS, whereas the earlier densification of X-CRS-10 slightly lowers its final value. The curves of X-CRS-20 and X-CRS-30 remain close over most of the strain range, but the larger *ε*_d_ of X-CRS-30 produces the highest final energy absorption and SEA, as summarized in Figure 5b and Table 4. Thus, plateau stress, densification strain, and effective crushing distance jointly control the final energy absorption.

The X-shaped ligaments increase stiffness and plateau capacity, while the final energy-absorption ranking also reflects densification strain and crushing distance. X-CRS-30 has the highest mean SEA within the tested range. Representative density descriptors, loading conditions, SEA evaluation limits, and SEA values from related additively manufactured cellular structures are summarized in Supplementary Table S1 for quantitative context. The associated deformation modes and *ν*_eff_ evolution are examined next.

### 3.2. Deformation Mechanism and Poisson’s Ratio Evolution

**Figure 6:**
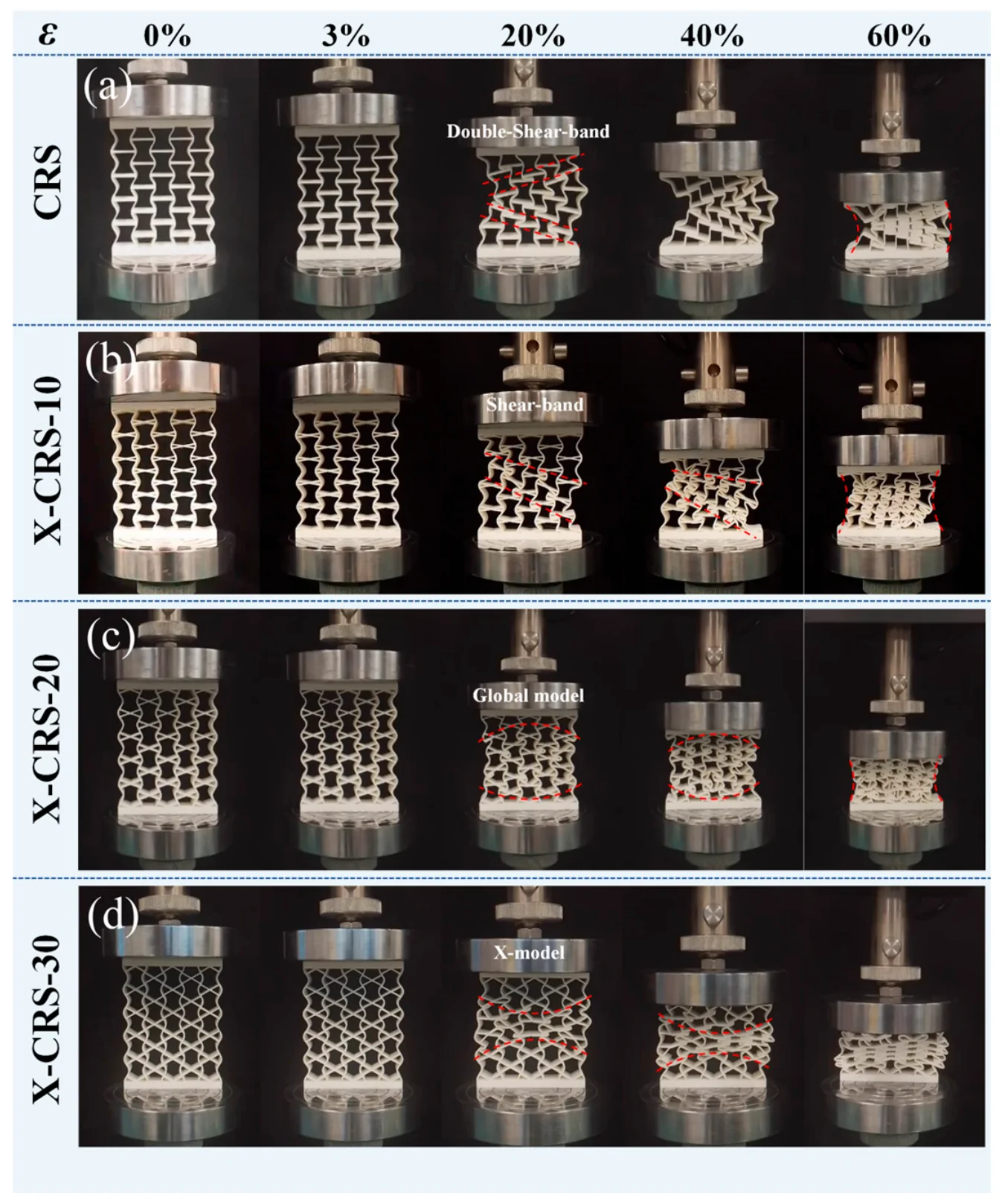
Deformation patterns at representative nominal compressive strains under quasi-static in-plane compression: (**a**) CRS; (**b**) X-CRS-10; (**c**) X-CRS-20; (**d**) X-CRS-30.

Following the differences in mechanical response and energy absorption discussed above, the deformation evolution was further examined to clarify how the X-shaped ligament angle affects collapse stability and auxetic behavior. Figure 6 presents the deformation patterns of the four structures at representative compressive strains. For CRS, a double-shear-band deformation is already observed at a strain of 20%, which subsequently develops into pronounced global tilting before densification. X-CRS-10 exhibits a single-shear-band collapse mode, but no obvious lateral buckling of the overall structure is observed during compression. By contrast, X-CRS-20 undergoes a more progressive deformation process, and the structural configuration remains relatively stable over a wide strain range. It is also noted that both X-CRS-10 and X-CRS-20 exhibit evident lateral contraction during compression, indicating a clear negative Poisson’s ratio response. X-CRS-30 shows a distinct X-shaped collapse mode, in which deformation initiates from the middle layer and gradually propagates to adjacent regions with increasing strain.

**Figure 7:**
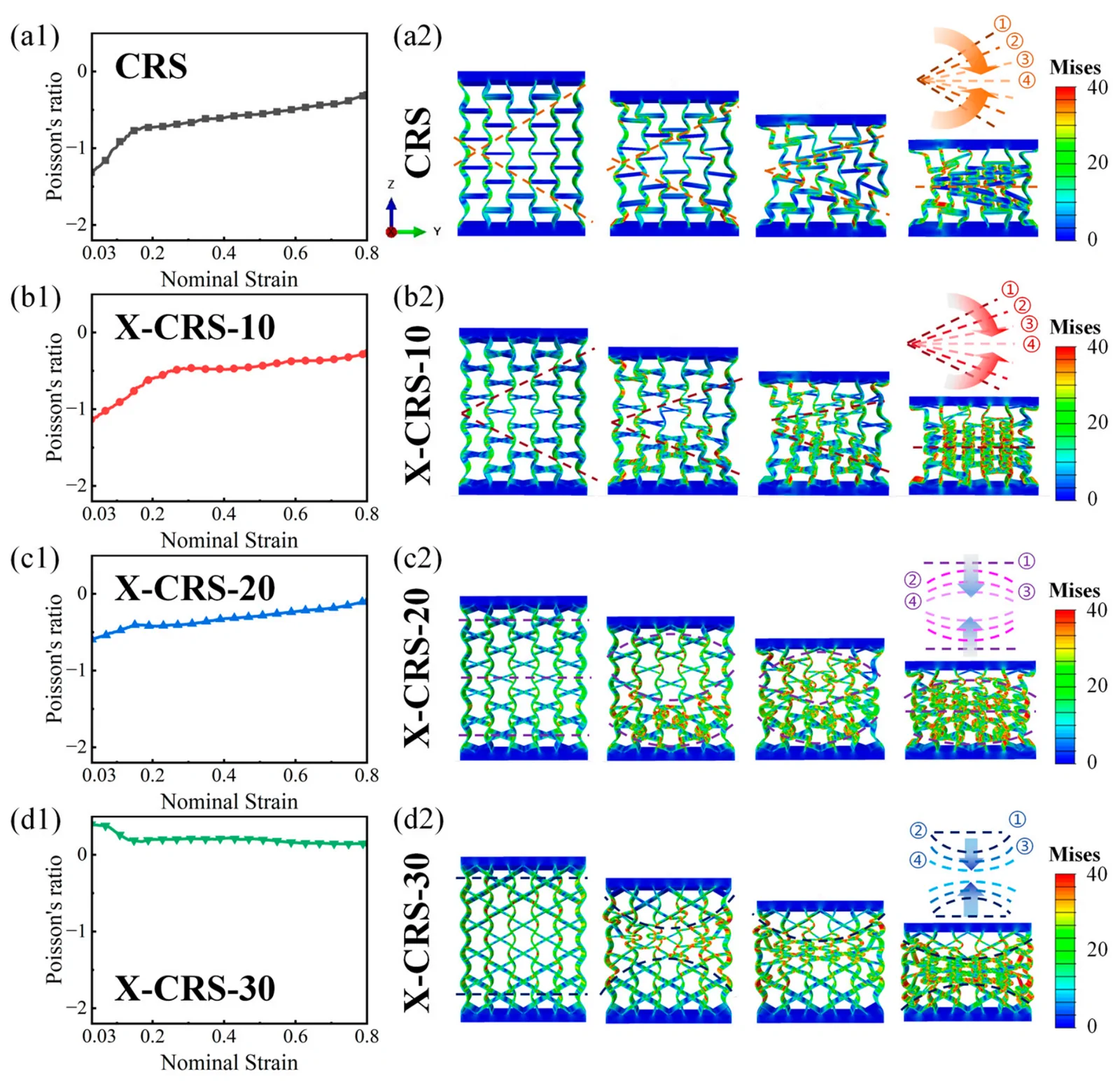
Numerical *ν*_eff_ evolution and corresponding finite element deformation processes of CRS and X-CRS structures under in-plane compression: (**a1**–**d1**) Poisson’s ratio–strain curves; (**a2**–**d2**) simulated deformation patterns at representative compressive strains. Numbers ①–④ indicate the compression sequence, and the von Mises stress contours are expressed in MPa.

To further quantify the auxetic response underlying the deformation modes discussed above, Figure 7 presents the numerical effective Poisson’s ratio, *ν*_eff_, evolution of each structure together with the corresponding finite element deformation process, where Figure 7(a1–d1) shows the *ν*_eff_–strain curves and Figure 7(a2–d2) illustrates the simulated deformation sequences. As shown in Figure 7(a1–d1), CRS exhibits the strongest negative Poisson’s ratio response at the early stage of compression, with *ν*_eff_ of approximately −1.2. The value then gradually increases to about −0.3 with increasing strain, indicating that its lateral contraction capability weakens during large deformation. X-CRS-10 still maintains a pronounced negative Poisson’s ratio response, with *ν*_eff_ increasing from approximately −1.1 to −0.3, and the curve varies more smoothly than that of CRS. As the included angle of the X-shaped ligaments increases to 20°, the *ν*_eff_ of X-CRS-20 further increases from approximately −0.6 toward zero, suggesting a weakened but still observable lateral contraction behavior throughout compression. In contrast, X-CRS-30 maintains a positive *ν*_eff_ during compression, indicating that this configuration no longer exhibits a typical negative Poisson’s ratio deformation mode. For a quantitative comparison at a representative nominal compressive strain of *ε*_c_ = 0.20, the numerical *ν*_eff_ values of CRS, X-CRS-10, X-CRS-20, and X-CRS-30 are −0.676, −0.609, −0.376, and +0.156, respectively. These values further confirm the progressive weakening of the auxetic response with increasing *θ* and the transition to a non-auxetic response in X-CRS-30.

The Poisson’s ratio evolution is consistent with the finite element deformation processes shown in Figure 7(a2–d2). At the initial compression stage, CRS generates lateral contraction mainly through cell-wall bending and unit-cell rotation, thereby exhibiting a relatively low Poisson’s ratio. With the progressive development of inclined high-stress bands, the structure undergoes shear-band-like collapse and global tilting, accompanied by a gradual increase in Poisson’s ratio. X-CRS-10 still shows evident lateral contraction, while the introduction of X-shaped ligaments suppresses global lateral buckling and changes the deformation mode from the double-shear-band collapse of CRS to a more concentrated single-shear-band mode. X-CRS-20 exhibits a more stable progressive collapse process, in which high-stress regions are distributed over multiple unit-cell regions and the overall configuration maintains better deformation compatibility over a wide strain range. As a result, a limited negative Poisson’s ratio response is retained. For X-CRS-30, collapse initiates from the middle region and then propagates to adjacent regions along the X-shaped ligament network, forming a typical X-shaped collapse mode. In this case, the X-shaped ligaments dominate the load-transfer and collapse paths, significantly suppressing the lateral contraction of the CRS unit cells and causing the Poisson’s ratio to change from negative to positive. As *θ* increases, the X-shaped ligaments provide a stronger load-transfer path while imposing a greater constraint on the rotation of the re-entrant walls. For X-CRS-30, this constraint becomes sufficiently strong to suppress the transverse contraction responsible for the auxetic response, while simultaneously contributing to its higher stiffness and energy-absorption capacity. Therefore, the positive *ν*_eff_ of X-CRS-30 reflects a transition in the dominant deformation mechanism rather than simply a quantitative reduction in auxeticity.

Overall, with increasing included angle of the X-shaped ligaments, the dominant deformation mechanism gradually shifts from CRS unit-cell rotation and lateral contraction to progressive collapse and load transfer regulated by the X-shaped ligament network. Among the investigated configurations, X-CRS-20 provides a better balance among negative Poisson’s ratio response, deformation stability, and load-transfer capability, whereas X-CRS-30 transforms into a non-auxetic collapse mode governed by the X-shaped ligament network. Accordingly, X-CRS-20 better preserves the auxetic deformation mechanism, whereas X-CRS-30 favors stiffness and energy absorption at the expense of auxeticity.

### 3.3. Scope and Limitations

The present study is limited to PolyMax PLA, one flat build orientation, three X-ligament angles (10°, 20°, and 30°), one specimen scale, quasi-static in-plane compression, and three experimental replicates per configuration. Therefore, the observed angle-dependent trends are not intended to identify a global optimum, and the limited sample size warrants treating the statistical analysis as exploratory. Because FDM properties are orientation dependent, the reported experimental response is specific to the adopted build orientation. The effective isotropic finite element model does not explicitly represent FDM-induced anisotropy, geometric imperfections, local material heterogeneity, interlayer damage, microcracking, or fracture; accordingly, it is used primarily to interpret global response trends, load-transfer paths, and deformation mechanisms rather than to predict fracture or claim exact pointwise agreement with the experiments. In addition, the cross-study SEA comparison in Supplementary Table S1 is intended to provide quantitative context rather than an absolute performance ranking because the referenced studies differ in material, density descriptor, loading condition, and SEA evaluation limit. Further validation under dynamic, impact, and fatigue loading, as well as across different materials, specimen scales, build orientations, and a broader X-ligament-angle range, is required before practical implementation.

## 4. Conclusions

In this study, a honeycomb with cosine-curved walls and X-shaped ligaments, denoted as X-CRS, was proposed to improve the in-plane mechanical response and energy absorption performance of conventional CRS structures. Quasi-static compression experiments and finite element simulations were conducted to evaluate the mechanical properties, deformation modes, energy absorption behavior, and Poisson’s ratio evolution. The main conclusions are summarized as follows:

(1) X-shaped ligaments improve load-bearing capacity. X-CRS-30 reached *E* = 16.76 MPa, *σ*_c_ = 0.512 MPa, and *σ*_pl_ = 0.422 MPa, corresponding to descriptive increases of approximately 148%, 133%, and 102% relative to CRS.
(2) Energy absorption depends on both plateau stress and densification strain. X-CRS-30 combined a high *σ*_pl_ with the largest mean *ε*_d_ and achieved the highest mean SEA, 1.225 ± 0.015 kJ kg^−1^, approximately 90% above CRS; earlier densification limited the final SEA of X-CRS-10.
(3) The deformation mode is strongly affected by the included angle of the X-shaped ligaments. CRS shows double-shear-band deformation and global tilting, while X-CRS-10 and X-CRS-20 exhibit improved deformation stability. X-CRS-20 presents a progressive collapse mode with better deformation compatibility, whereas X-CRS-30 develops an X-shaped collapse mode initiated from the middle region.
(4) The evolution of *ν*_eff_ reveals a transition in the dominant deformation mechanism. CRS, X-CRS-10, and X-CRS-20 retain negative Poisson’s ratio responses to varying degrees, whereas X-CRS-30 shifts to a non-auxetic but more load-bearing collapse mode. X-CRS-20 provides a better balance between auxeticity and collapse stability, while X-CRS-30 favors higher stiffness and energy absorption at the expense of auxeticity. Therefore, neither configuration can be regarded as universally optimal.

## Figures and Tables

**Table 1:** Geometric parameters of the CRS and X-CRS specimens.

Structure	*θ* (°)	Attachment Point *P*(*x*_a_, *y*_a_) (mm)	Full X-Ligament Length, *L*_X_ (mm)
CRS	—	—	—
X-CRS-10	10	(9.72, 1.71)	19.74
X-CRS-20	20	(9.03, 3.29)	19.22
X-CRS-30	30	(8.18, 4.72)	18.89

Note: The common geometric parameters are *l* = 20 mm, *h* = 20 mm, *t* = 1 mm, *T* = 6 mm, and *W* × *H* × *D* = 85 × 112 × 20 mm. The X-ligament thickness was 1.0 mm for all X-CRS configurations and is not applicable to CRS. To minimize relative-density effects, the CRS horizontal-ligament thickness was set to 1.82 mm, giving relative-volume differences of 0.018%, 0.591%, and 0.241% with respect to X-CRS-10, X-CRS-20, and X-CRS-30, respectively. *O*(0, 0) denotes the unit-cell center; *P*(*x*_a_, *y*_a_) denotes the upper-right attachment point, with the other attachment points following centrosymmetry; *L*_X_ denotes the full X-ligament length.

**Table 2:** FDM processing parameters used for the PolyMax PLA honeycomb specimens.

Processing Parameter	Value or Setting
Printer and software	Bambu Lab H2D (Shenzhen Tuozhu Technology Co., Ltd., Shenzhen, China); Bambu Studio v02.06.01.55
Material and nozzle	PolyMax PLA, 1.75 mm; hardened-steel nozzle, 0.40 mm
Build orientation	Flat; honeycomb plane parallel to build plate; layer stacking through thickness
Layer height and line width	0.10 mm nominal; 0.20 mm initial; 0.35 mm line width
Nozzle/build-plate temperature	220/55 °C
Printing speeds	Initial perimeter/infill 20/40; outer/inner wall 35/80; infill/top 80/60 mm s^−1^
Infill	100%; zig-zag; 45°
Walls and solid layers	5 wall loops; 5 top and 3 bottom solid layers
Support and adhesion	Support disabled; automatic 8 mm brim

**Table 3:** Key finite element settings and verification results.

Model Item	Implemented Setting
Solver and mesh	Abaqus/Explicit; C3D8R; two analytical rigid platens; production mesh = 0.33 mm
Material	*E* = 1370 MPa; *ν* = 0.30; *ρ* = 1240 kg m^−3^; coupon-derived elastoplastic hardening
Contact	General Contact with ALL EXTERIOR; wall self-contact; hard normal contact; *μ* = 0.10
Boundary and loading	Lower platen fixed; upper platen laterally constrained and prescribed to *U*_3_ = −100 mm over 0.1 s
Mesh verification	0.50, 0.33, and 0.25 mm meshes; production-to-fine differences ≤ 5.96%; 0.33 mm mesh adopted
Quasi-static verification	No artificial mass scaling; ALLKE/ALLIE monitored; <1.6% for *ε* ≥ 0.05

Note: For the 0.33 mm production meshes, the node/element counts were 1,711,450/1,461,440 for CRS, 1,876,122/1,560,260 for X-CRS-10, 1,760,802/1,436,003 for X-CRS-20, and 1,847,850/1,524,209 for X-CRS-30.

**Table 4:** Experimental mechanical and energy-absorption metrics (mean ± sample standard deviation; *n* = 3), together with numerical *ν*_eff_ at *ε*_c_ = 0.20.

Structure	*E* (MPa)	*σ*_c_ (MPa)	*σ*_pl_ (MPa)	*ε*_d_ (mm/mm)	*W*(*ε*_d_) (MJ m^−3^)	SEA(*ε*_d_) (kJ kg^−1^)	Numerical *ν*_eff_ (*ε*_c_ = 0.20)
CRS	6.768 ± 0.105	0.220 ± 0.017	0.209 ± 0.035	0.491 ± 0.104	0.143 ± 0.004	0.644 ± 0.003	−0.676
X-CRS-10	9.150 ± 0.936	0.297 ± 0.015	0.268 ± 0.029	0.420 ± 0.081	0.135 ± 0.011	0.608 ± 0.059	−0.609
X-CRS-20	13.72 ± 0.371	0.455 ± 0.030	0.392 ± 0.023	0.422 ± 0.037	0.207 ± 0.017	0.909 ± 0.076	−0.376
X-CRS-30	16.76 ± 0.243	0.512 ± 0.010	0.422 ± 0.045	0.620 ± 0.006	0.268 ± 0.007	1.225 ± 0.015	+0.156

## Data Availability

The original contributions presented in this study are included in the article/Supplementary Material. Further inquiries can be directed to the corresponding author.

## Supplementary Materials

The following supporting information can be downloaded at: https://www.mdpi.com/article/10.3390/ma19163533/s1. Note S1: Mesh-Sensitivity Assessment; Note S2: Quasi-Static Verification; Note S3: Post-Test Damage Observation; Note S4: Cross-Study SEA Comparison; Figure S1: Mesh-sensitivity assessment for X-CRS-20; Figure S2: ALLKE/ALLIE histories for the four 0.33 mm production models; Figure S3: Post-test photographs of highly deformed specimens; Table S1: Density descriptors and specific energy absorption of the present CRS/X-CRS configurations and representative additively manufactured cellular structures under quasi-static compression. Refs [29,30,31,32] are included in Supplementary Materials.

## References

[1] Chen Y., Pan Z., Qin J., Wang Y., Lin C. (2026). Novel auxetic hybrid honeycombs with enhanced design flexibility and mechanical properties. Compos. Struct..

[2] Chen Z., Li J., Wu B., Chen X., Xie Y.M. (2024). Enhanced mechanical properties of re-entrant auxetic honeycomb with self-similar inclusion. Compos. Struct..

[3] Hu L.L., Zhou M.Z., Deng H. (2019). Dynamic indentation of auxetic and non-auxetic honeycombs under large deformation. Compos. Struct..

[4] Shao Q., Ding C., Ji X., Mu J., Wang X., Xue Y. (2024). Fabrication, microstructure and mechanical properties of a 3D re-entrant anti-trichiral honeycomb structure with excellent auxeticity and mechanical performance. J. Mater. Res. Technol..

[5] Kadic M., Milton G.W., van Hecke M., Wegener M. (2019). 3D metamaterials. Nat. Rev. Phys..

[6] Jiao P., Mueller J., Raney J.R., Zheng X., Alavi A.H. (2023). Mechanical metamaterials and beyond. Nat. Commun..

[7] Frenzel T., Hahn V., Ziemke P., Schneider J.L.G., Chen Y., Kiefer P., Gumbsch P., Wegener M. (2021). Large characteristic lengths in 3D chiral elastic metamaterials. Commun. Mater..

[8] Montgomery-Liljeroth E., Schievano S., Burriesci G. (2023). Elastic properties of 2D auxetic honeycomb structures- a review. Appl. Mater. Today.

[9] Hameed H.M., Hasan H.M. (2025). Exploring honeycomb structures: A review of their types, general applications, and role in vibration damping and structural stability. Structures.

[10] Cai Z., Deng X. (2026). Recent advances in reinforced honeycomb mechanical metamaterials: A review. Mater. Des..

[11] Xue M.L., Zhang Y., Teng X.C., Jiang W.Z., Xue T., Qu Y.C., Ji X., Shen C., Ren X. (2025). Multifunctional auxetic re-entrant metastructure for low-frequency broadband sound absorption and energy absorption. Thin Walled Struct..

[12] Xu Z.H., Cui Y.J., Zhou Q., Wang K.F., Wang B. (2025). Three-point bending performance of sandwich beam with novel gradient re-entrant-chiral-triangular honeycomb core. Eng. Struct..

[13] Ma N., Han Q., Han S., Li C. (2023). Hierarchical re-entrant honeycomb metamaterial for energy absorption and vibration insulation. Int. J. Mech. Sci..

[14] Li R., Zhong Y., Tang Y., Liu R. (2025). Enhanced specific stiffness and energy absorption in star-re-entrant hierarchical honeycombs featuring two plateau stages. Thin Walled Struct..

[15] Liu J., Liu J., Gao K., Mohagheghian I., Fan W., Yang J., Wu Z. (2025). A bioinspired gradient curved auxetic honeycombs with enhanced energy absorption. Int. J. Mech. Sci..

[16] Xia P., Li N., Fu H., Wang L., Qin H., Xiong C., Yu X., Wang Q., Wang C., Zhao F. (2024). Achieving high strength and energy absorption of novel 3D printed helical layered square honeycombs. Thin Walled Struct..

[17] Xia P., Liu Q., Fu H., Yu Y., Wang L., Wang Q., Yu X., Zhao F. (2023). Mechanical properties and energy absorption of 3D printed double-layered helix honeycomb under in-plane compression. Compos. Struct..

[18] Wang W.-J., Zhang W.-M., Yang H., Li Z.-Y., Ma L. (2026). Energy absorption characteristics of embedded mechanical metamaterials. Int. J. Mech. Sci..

[19] Wang H., Sun Y., Lan X., Zhao H., Yu Y.-H., Huang W., Liu Y., Leng J. (2025). Reconfigurable metamaterial honeycomb sandwich panels based on embedded tube Helmholtz resonators. Compos. Struct..

[20] Cai S., Lai Y., Kong H., Zhong B., Chen J., Zhang Y. (2026). Hybrid honeycombs with enhanced in-plane compressive strength and tailored Poisson’s ratio. Constr. Build. Mater..

[21] Yuan X., Chen M., Yao Y., Guo X., Huang Y., Peng Z., Xu B., Lv B., Tao R., Duan S. (2021). Recent progress in the design and fabrication of multifunctional structures based on metamaterials. Curr. Opin. Solid State Mater. Sci..

[22] Feng J., Liang Q., Dou Y., He J., Wu Y., Chen T. (2022). Higher stiffness hierarchical embedded strengthening honeycomb metastructure with small negative Poisson’s ratio reduction. Thin Walled Struct..

[23] Wang Y., Ren Y., Jiang H. (2025). A bionic concentric honeycomb with synergetic enhancement mechanism. Thin Walled Struct..

[24] Montazeri A., Mahnama M. (2025). Twisting chiral mechanical metamaterials: A review. Mater. Today Commun..

[25] Zhang C., Lu F., Mo K., He Y., Liu Y., Zhu Y. (2026). Coupling negative Poisson’s ratio with multifunctionality: Advances in auxetic metamaterials. Int. J. Solids Struct..

[26] Jiao C., Yan G. (2021). Design and elastic mechanical response of a novel 3D-printed hexa-chiral helical structure with negative Poisson’s ratio. Mater. Des..

[27] Zhang Z., Zhang L.A., Dong Y., Chen H., Guo Y. (2023). Mechanical properties of negative Poisson’s ratio metamaterial units and honeycomb structures with cosine-like re-entrant structure. Mater. Lett..

[28] Cheng X., Zhang Y., Ren X., Han D., Jiang W., Zhang X.G., Luo H.C., Xie Y.M. (2022). Design and mechanical characteristics of auxetic metamaterial with tunable stiffness. Int. J. Mech. Sci..

[29] Overbeck M., Heimbs S., Kube J., Hühne C. (2024). Energy Absorption Properties of 3D-Printed Polymeric Gyroid Structures for an Aircraft Wing Leading Edge. Aerospace.

[30] Novak N., Grebo A., Borovinšek M., Krstulović-Opara L., Ren Z., Vesenjak M. (2025). Deformation Behaviour of Optimised Three-Dimensional Axisymmetric Chiral Auxetic Structures. Biomedicines.

[31] Panowicz R., Jeschke A., Durejko T., Zachman M., Konarzewski M. (2024). The Mechanical Properties and Energy Absorption of AuxHex Structures. Materials.

[32] Ghimire A., Hsu C.-H., Lin C.-C., Chen P.-Y. (2024). Hierarchical nested honeycomb-based energy absorbers: Design factors and tailorable mechanical properties. Interface Focus.

