# Supplementary material for: A Novel Re-Entrant Honeycomb with Tunable Auxetic Response and Enhanced In-Plane Mechanical Performance
Source: Materials (Basel). 2026 Aug 20;19(16):3533. doi: 10.3390/ma19163533 (PMC13514213; doi:10.3390/ma19163533)
Supplement: Supplementary file 1 [file materials-19-03533-s001.zip › materials-4455158-supplementary.pdf]

Supplementary Information

A Novel Re-Entrant Honeycomb with Tunable Auxetic Response and Enhanced In-Plane Mechanical Performance

This file includes:

Supplementary Notes S1 to S4

Figures S1 to S3

Table S1

Supplementary References

Supplementary Note S1. Mesh-Sensitivity Assessment

Mesh sensitivity was evaluated for X-CRS-20 using global element sizes of 0.50, 0.33, and 0.25 mm. Relative to the 0.25 mm reference, the 0.33 mm production mesh differed by 3.68% in full-curve L_2 norm, 1.80% in collapse stress, 1.54% in plateau stress, 0.81% in SEA, 2.31% in densification strain, and 5.96% in the initial-modulus diagnostic. The ν_{eff} curve differed by 2.46% between the 0.33 and 0.25 mm meshes; therefore, 0.33 mm was adopted.

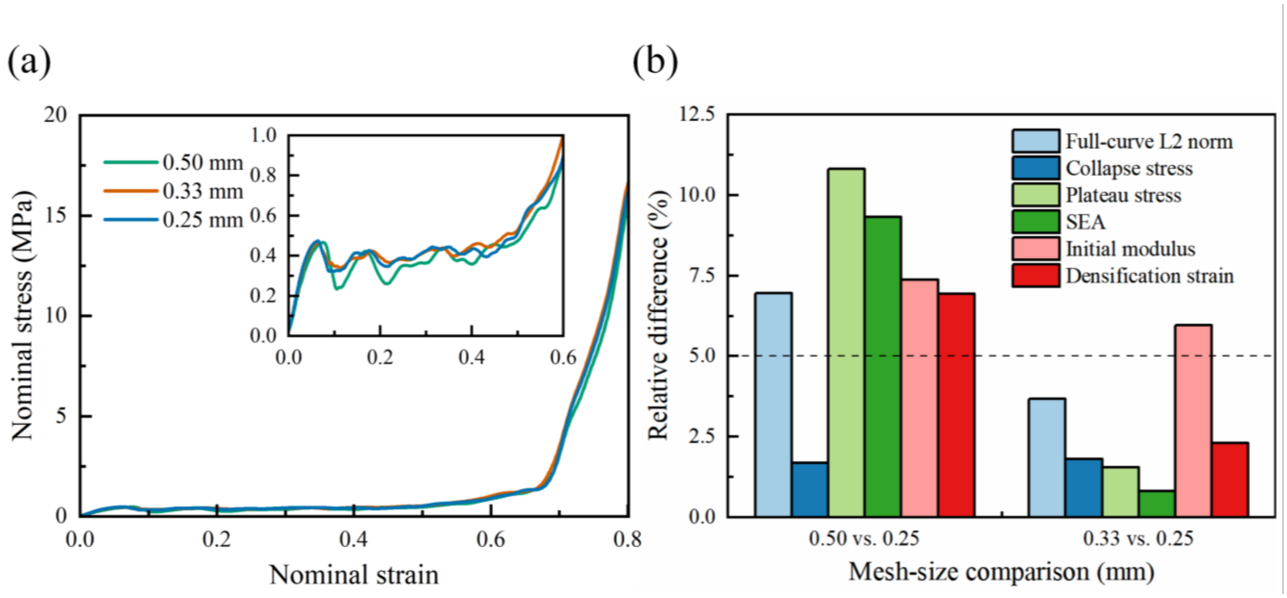

Figure S1. Mesh-sensitivity assessment for X-CRS-20: (a) nominal stress–strain responses for 0.50, 0.33, and 0.25 mm meshes; (b) relative differences with respect to the 0.25 mm reference. The dashed line indicates the 5% reference criterion.

Supplementary Note S2. Quasi-Static Verification

The kinetic-to-internal-energy ratio (ALLKE/ALLIE) was monitored without artificial mass scaling. Over the full $0.02 \leq \varepsilon \leq 0.60$ histories, the maximum ratios were 5.69%, 8.08%, 2.23%, and 2.37% for CRS, X-CRS-10, X-CRS-20, and X-CRS-30, respectively; after $\varepsilon = 0.05$, all ratios remained below 1.6%. These histories support use of the Explicit solutions for quasi-static trend and mechanism interpretation.

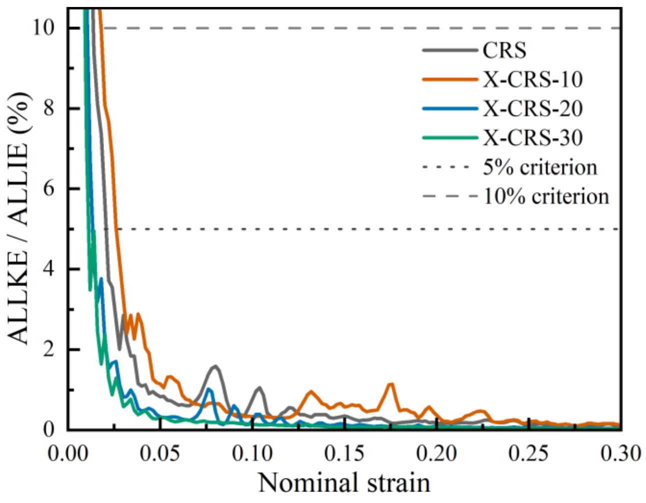

Figure S2. ALLKE/ALLIE histories for the four 0.33 mm production models. The plot shows $0 \le \varepsilon \le 0.30$ with 5% and 10% reference levels; reported maxima were evaluated over $0.02 \le \varepsilon \le 0.60$.

Supplementary Note S3. Post-Test Damage Observation

Post-test photographs of the highly deformed CRS and X-CRS-30 specimens shown in Figure S3(a, b) were examined to assess the experimental basis for omitting a damage-evolution law. No visible macroscopic cracking or ligament fracture was observed in the photographed regions, although the images cannot exclude microcracking. The finite element model is therefore limited to global response and deformation mechanisms and is not used to predict crack initiation or fracture.

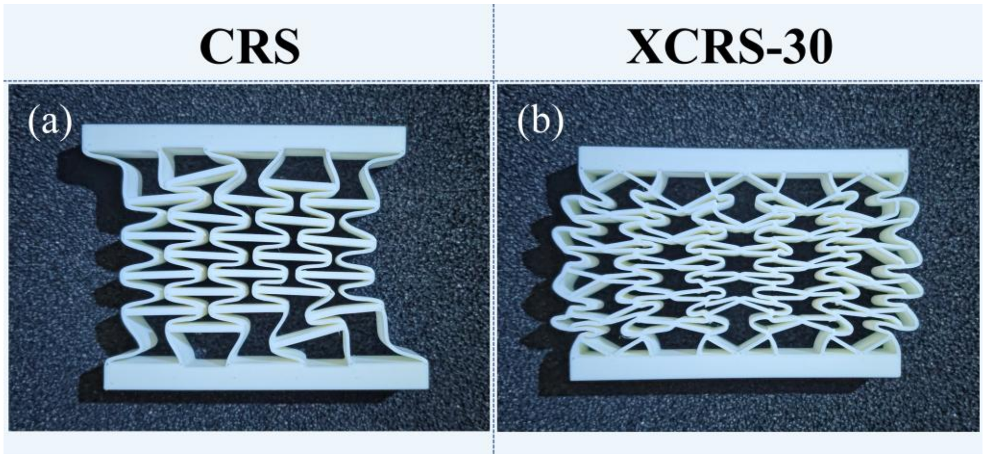

Figure S3. Post-test photographs of highly deformed specimens: (a) CRS; (b) X-CRS-30. No visible macroscopic cracking or ligament fracture was observed in the photographed regions.

Supplementary Note S4. Cross-Study SEA Comparison

Table S1 provides quantitative context for the SEA performance of the present CRS/X-CRS configurations relative to representative additively manufactured cellular structures reported in the literature. Because the compared studies differ in material, manufacturing process, density descriptor, loading condition, and SEA evaluation limit, the values are intended to provide quantitative context rather than an absolute performance ranking.

[a] The four present configurations were designed with nominally matched relative volumes; the maximum difference was 0.591%. Density was therefore treated as a control condition rather than an explanatory variable.

[b] SEA values for Xia et al. [1] were reconstructed from the fitted relationships reported in that study and are not independently tabulated measurements.

[c] The range for Overbeck et al. [2] spans the available unit-cell sizes at the stated relative densities; only quasi-static data were retained, and SEA was evaluated at 50% compression.

[d] Values for Novak et al. [3] were approximately digitized from Figure 6; the two values correspond to strains of 0.40 and 0.60.

[e] For Panowicz et al. [4], SEA is reported as mean ± standard deviation. The envelope density was estimated from the reported absorbed energy, SEA, and specimen dimensions and is not relative density.

[f] Relative density and SEA values for Ghimire et al. [5] were transcribed from the numerical labels in Figure 4d.

Table S1. Density descriptors and specific energy absorption of the present CRS/X-CRS configurations and representative additively manufactured cellular structures under quasi-static compression.

Study	Structure or series	Material / process	Loadin g conditio n	Density descriptor	SEA evaluation limit	SEA (kJ kg^{-1})	References

Study	Structure or series	Material / process	Loading condition	Density descriptor	SEA evaluation limit	SEA (kJ kg^{-1})	References
Present study	CRS	PolyMax PLA / FDM	In-plane quasi-static	Nominally matched[a]	Densification strain	0.644 ± 0.003	This work
	X-CRS-10	PolyMax PLA / FDM	In-plane quasi-static	Nominally matched[a]	Densification strain	0.608 ± 0.059	This work
	X-CRS-20	PolyMax PLA / FDM	In-plane quasi-static	Nominally matched[a]	Densification strain	0.909 ± 0.076	This work
	X-CRS-30	PolyMax PLA / FDM	In-plane quasi-static	Nominally matched[a]	Densification strain	1.225 ± 0.015	This work
Xia et al. (2023)	Regular honeycomb (RH)	PolyMax PLA / FFF	In-plane quasi-static	ρ_r = 14/17/21/26 %	Densification strain	1.408/1.802/2.356/3.090[b]	[1]
	Double-layered helix honeycomb (DLHH)	PolyMax PLA / FFF	In-plane quasi-static	ρ_r = 14/17/21/26 %	Densification strain	4.038/4.705/5.558/6.576[b]	[1]
Overbeck et al. (2024)	Sheet-gyroid; cell size 7–20 mm	Standard Resin / SLA	Quasi-static	ρ_r = 7–23%	50% compression	1.05–4.40[c]	[2]
	Sheet-gyroid; cell size 7–20 mm	PU Rigid 650 / SLA	Quasi-static	ρ_r = 23%	50% compression	1.34–3.15[c]	[2]
	Sheet-gyroid; cell size 14–20 mm	Nylon 11 / SLS	Quasi-static	ρ_r = 7–23%	50% compression	1.48–7.96[c]	[2]
Novak et al. (2025)	Axisymmetric chiral structure (ACS)	Photopolymer / AM	Quasi-static; 0.1 mm s−1	ρ_r = 9.67%	40% / 60% strain	0.52/0.89[d]	[3]
	Optimized ACS (optACS)	Photopolymer / AM	Quasi-static; 0.1 mm s−1	ρ_r = 20.15%	40% / 60% strain	1.18/1.95[d]	[3]
Panowicz et al. (2024)	AuxHex a, *x*-direction	ULTEM 9085 (PEI) / HT-FFF	In-plane quasi-static	ρ_{env} ≈ 0.306 g cm−3	Densification strain	7.60 ± 1.38[e]	[4]
	AuxHex a,	ULTEM	In-plane	ρ_{env} ≈ 0.329	Densificati	3.03 ± 0.07[e]	[4]

Study	Structure or series	Material / process	Loading condition	Density descriptor	SEA evaluation limit	SEA (kJ kg^{-1})	References
	y-direction	9085 (PEI) / HT-FFF	quasi-static	g cm−3	on strain		
	AuxHex b, *x*-direction	ULTEM 9085 (PEI) / HT-FFF	In-plane quasi-static	ρ_{env} ≈ 0.314 g cm−3	Densification strain	6.33 ± 0.83[e]	[4]
	AuxHex b, *y*-direction	ULTEM 9085 (PEI) / HT-FFF	In-plane quasi-static	ρ_{env} ≈ 0.335 g cm−3	Densification strain	6.13 ± 0.31[e]	[4]
Ghimire et al. (2024)	Hierarchical nested honeycomb I0	TPU / FDM	In-plane quasi-static	ρ_r = 15%	As defined in Fig. 4	0.091[f]	[5]
	Hierarchical nested honeycomb I3	TPU / FDM	In-plane quasi-static	ρ_r = 23%	As defined in Fig. 4	0.173[f]	[5]
	Hierarchical nested honeycomb I4	TPU / FDM	In-plane quasi-static	ρ_r = 25%	As defined in Fig. 4	0.178[f]	[5]
	Hierarchical nested honeycomb I5	TPU / FDM	In-plane quasi-static	ρ_r = 27%	As defined in Fig. 4	0.403[f]	[5]
	Hierarchical nested honeycomb I7	TPU / FDM	In-plane quasi-static	ρ_r = 30%	As defined in Fig. 4	0.456[f]	[5]

Note: AM, additive manufacturing; FDM/FFF, fused deposition modeling/fused filament fabrication; HT-FFF, high-temperature FFF; PEI, polyetherimide; PU, polyurethane; SLA, stereolithography; SLS, selective laser sintering. 1 J g^{-1} = 1 kJ kg^{-1}.

Supplementary References

[1] P. Xia, Q. Liu, H. Fu, Y. Yu, L. Wang, Q. Wang, X. Yu, F. Zhao, Mechanical properties and energy absorption of 3D printed double-layered helix honeycomb under in-plane compression, Composite Structures 315 (2023) 116982. https://doi.org/10.1016/j.compstruct.2023.116982.

[2] M. Overbeck, S. Heimbs, J. Kube, C. Hühne, Energy absorption properties of 3D-printed polymeric gyroid structures for an aircraft wing leading edge, Aerospace 11 (2024) 801. https://doi.org/10.3390/aerospace11100801.

[3] N. Novak, A. Grebo, M. Borovinšek, L. Krstulović-Opara, Z. Ren, M. Vesenjak, Deformation behaviour of optimised three-dimensional axisymmetric chiral auxetic structures, Biomedicines 13 (2025) 2816. https://doi.org/10.3390/biomedicines13112816.

[4] R. Panowicz, A. Jeschke, T. Durejko, M. Zachman, M. Konarzewski, The mechanical properties and energy absorption of AuxHex structures, Materials 17 (2024) 6073. https://doi.org/10.3390/ma17246073.

[5] A. Ghimire, C.-H. Hsu, C.-C. Lin, P.-Y. Chen, Hierarchical nested honeycomb-based energy absorbers: design factors and tailorable mechanical properties, Interface Focus 14 (2024) 20230066. https://doi.org/10.1098/rsfs.2023.0066.
